# SIRT1: Genetic Variants and Serum Levels in Age-Related Macular Degeneration

**DOI:** 10.3390/life12050753

**Published:** 2022-05-19

**Authors:** Kriste Kaikaryte, Greta Gedvilaite, Alvita Vilkeviciute, Loresa Kriauciuniene, Ruta Mockute, Dzastina Cebatoriene, Reda Zemaitiene, Vilma Jurate Balciuniene, Rasa Liutkeviciene

**Affiliations:** 1 Laboratory of Ophthalmology, Neuroscience Institute, Medical Academy, Lithuanian University of Health Sciences, Eiveniu 2, LT-50161 Kaunas, Lithuania; greta.gedvilaite@lsmuni.lt (G.G.); alvita.vilkeviciute@lsmuni.lt (A.V.); loresa.kriauciuniene@lsmuni.lt (L.K.); rasa.liutkeviciene@lsmuni.lt (R.L.); 2 Department of Ophthalmology, Medical Academy, Lithuanian University of Health Sciences, Eiveniu 2 Str., LT-50161 Kaunas, Lithuania; rutikess@gmail.com (R.M.); dzastina.cebatoriene@lsmuni.lt (D.C.); reda.zemaitiene@lsmuni.lt (R.Z.); jurate.balciuniene@lsmu.lt (V.J.B.)

**Keywords:** age-related macular degeneration (AMD), *SIRT1*, rs3818292, rs3758391, rs7895833, SIRT1 levels

## Abstract

**Background:** The aim of this paper was to determine the frequency of *SIRT1* rs3818292, rs3758391, rs7895833 single nucleotide polymorphism genotypes and SIRT1 serum levels associated with age-related macular degeneration (AMD) in the Lithuanian population. **Methods:** Genotyping of *SIRT1* rs3818292, rs3758391 and rs7895833 was performed using RT-PCR. SIRT1 serum level was determined using the ELISA method. **Results:** We found that rs3818292 and rs7895833 were associated with an increased risk of developing exudative AMD. Additional sex-differentiated analysis revealed only rs7895833 was associated with an increased risk of developing exudative AMD in women after strict Bonferroni correction. The analysis also revealed that individuals carrying rs3818292, rs3758391 and rs7895833 haplotype G-T-G are associated with increased odds of exudative AMD. Still, the rare haplotypes were associated with the decreased odds of exudative AMD. After performing an analysis of serum SIRT1 levels and *SIRT1* genetic variant, we found that carriers of the *SIRT1* rs3818292 minor allele G had higher serum SIRT1 levels than the AA genotype. In addition, individuals carrying at least one *SIRT1* rs3758391 T allele also had elevated serum SIRT1 levels compared with individuals with the wild-type CC genotype. **Conclusions:** Our study showed that the *SIRT1* polymorphisms rs3818292 and rs7895833 and rs3818292-rs3758391-rs7895833 haplotype G-T-G could be associated with the development of exudative AMD. Also, two SNPs (rs3818292 and rs3758391) are associated with elevated SIRT1 levels.

## 1. Introduction

Age-related macular degeneration (AMD) is presented as multifactorial ocular pathology and one of the most common causes of vision loss in the developed countries in people over 60 years of age [1,2]. According to the Lithuanian Commission for Medical-Social Expertise, AMD-related blindness accounts for 13.8% of all cases of blindness in the Lithuanian population [3]. The development of the disease begins with early AMD, which is defined by the presence of drusen and the retinal pigment epithelium (RPE) abnormalities. Early AMD transitions to advanced AMD; dry AMD, characterized by geographic atrophy of the RPE in the absence of neovascular AMD; or neovascular AMD, characterized by detachment of the retinal pigment epithelium, hemorrhaging, and scarring [4].

Numerous factors take part in the progress of this disease, including the body’s ageing processes and the pathological changes necessary for the development of this disease. Such processes include inflammation, changes in the extracellular matrix, oxidative stress, alterations in the biological activity of the retinal pigment epithelium, and genetic factors [5].

The sirtuins (SIRT) are known as a family of highly conserved class III NAD-dependent deacetylases. They contribute to the lifespan regulation processes of numerous organisms [6]. In mammals, seven human Sir2 homologs (sirtuins) have been identified to date, designated SIRT1 through SIRT7.

They strongly correlate with caloric limitation, ageing, processes of carcinoma, responses to stress, cellular alteration, metabolic function, inflammation, transcriptional silencing, chromosome stability, programmed cell death, DNA reparation, and prevention of age-related eye diseases. Generally, it is known that sirtuins may have an essential function in cellular senescence, cell alteration, and inflammation [7,8,9,10,11,12].

The human retina is the primary location where all sirtuins except SIRT5 are expressed [13,14]. It is a photoreceptive tissue

Its energy consumption varies based on the incidence of light. Retina tissue cells absorb extra energy during the dark time since they consume more oxygen and produce more lactate [15,16,17]. Therefore, the retinal expression of sirtuins varies, underlining the *regulatory* mechanisms of sirtuins in the retina.

It has been known that SIRT1 plays a part in forming new blood cells in vitro in zebrafish and mice. During the study, Potente et al. found that expression of SIRT1 raised during the mouse vasculature angiogenesis process. SIRT1 controls the angiogenic activity of mouses endothelial cells. In addition, loss of function studies has shown that processes of sprouting angiogenesis and branching morphogenesis of human endothelial cells were blocked. It resulted in the downregulation of genes required in angiogenesis and vascular remodeling. Disturbances of *SIRT1 expression* in the above-presented animals were followed by impaired angiogenesis and attenuation of the *ischemia-inducted* formation of new blood cells [18].

In recent years, it has been suggested that blocking the neovascularization chain may inhibit the development of AMD [19,20]. Therefore, we investigate the *SIRT1* rs3818292, rs3758391, and rs7895833 gene polymorphisms and SIRT1 serum levels that play a role in neovascularization of animal models.

*SIRT1* rs3818292 is located in the intronic region [21]. It is known that intronic variants are responsible for altering gene splicing processes [22]. *SIRT1* rs3758391 and rs7895833 are located in the promoter region [23]. This region is in charge of the initiation of gene transcription [24]. Due to this information, we have decided to focus on *SIRT1* rs3818292, rs3758391, and rs7895833. Characteristics of polymorphisms are provided in Table 1.

## 2. Materials and Methods

### 2.1. Ethics Statement

All subjects have signed an agreement according to the Declaration of Helsinki. The study was managed in the Department of Ophthalmology, Lithuanian University of Health Sciences. A total of 944 subjects were studied, and two study groups were formed: the control group (*n* = 225) and the group of patients with age-related macular degeneration (*n* = 719). Patient groups were divided into two subgroups, including patients with early AMD (*n* = 308) and patients with exudative AMD (*n* = 411). The control group comprised subjects who had no ocular pathology at examination and agreed to participate in this research.

Exclusion criteria of patients for the study were: (i) having an ocular disease unrelated to the study, for example, cloudy cornea, lens opacities (nuclear, cortical, or posterior subcapsular cataract) other than minor opacities, high refractive error, optic nerve disease, glaucoma, acute or chronic uveitis or keratitis; (ii) systemic diseases, e.g., malignant tumors, systemic connective tissue diseases, diabetes mellitus, chronic infections, or conditions following organ or tissue transplantation; and (iii) unevaluated color fundus photographs due to obscuration of the ocular optical system or quality of the fundus photograph.

Ophthalmologic examination of all subjects in our study was performed as previously described [27].

### 2.2. Ophthalmologic Evaluation

All subjects were tested by slit-lamp biomicroscopy to assess corneal and lens transparency. Lens opacities were classified and graded according to the Lens Opacities Classification System III. Intraocular pressure was measured at each examination. Pupils were dilated with tropicamide 1%. Fundoscopy with a direct monocular ophthalmoscope and slit-lamp biomicroscopy with a +78 diopter double aspheric lens was then performed. For more accurate analysis of the macula, stereoscopic color fundus photographs of the macula were taken at 45° and 30° to the fovea using a Visucam NM digital camera (Carl Zeiss Meditec AG, Jena, Germany).

All AMD patients experienced optical coherence tomography (OCT), and patients with suspected late AMD undertook fluorescein angiography after the examination OCT. For this study, we used the classification system for AMD, which was formulated by the previous Age-Related Eye Disease Study [4]: early AMD consisted of multiple small drusen and multiple intermediate (63–124 μm diameter) drusen or retinal pigment epithelial abnormalities. The presence of extensive intermediate drusen characterized early intermediate AMD and at least one large (≥125 μm diameter) drusen or geographic atrophy that did not involve the center of the fovea. Advanced AMD was characterized by geographic atrophy affecting the fovea and neovascular AMD features [4].

### 2.3. DNA Extraction, Genotyping and SIRT1 Serum Level Determination

DNA extraction and analysis of *SIRT1* rs3818292, rs3758391, and rs7895833 were carried out at the Laboratory of Ophthalmology, Neuroscience Institute, Lithuanian University of Health Sciences. DNA extraction was carried out using the DNA salting-out method from venous blood samples (white blood cells).

All single nucleotide polymorphisms (SNPs) were determined using TaqMan^®^ genotyping assays (Thermo Scientific, Pleasanton, CA, USA). Genotyping of *SIRT1* rs3818292, rs3758391 and rs7895833 was conducted using real-time PCR according to the manufacturer’s recommendations using a Step One Plus real-time PCR system (Applied Biosystems, Foster City, CA, USA). Poper real-time PCR mixtures *SIRT1* rs3818292, rs3758391, and rs7895833 were produced for SNP determination.

To every 96 wells of the plate, we added a PCR reaction mixture (8.5 μL) and 1.5 μL of the samples’ DNA. In addition, a negative control was added. We used the Allelic Discrimination program during a real-time PCR. Later, the assay was carried on according to the manufacturer’s instructions. The program analyzed the individual genotypes based on the different detectors’ fluorescence intensity (VIC and FAM).

Serum SIRT1 levels were calculated in control subjects and patients using enzyme-linked immunosorbent assay (ELISA) for human SIRT1 (Human SIRT1 ELISA Kit, Abcam, Cambridge, UK). Measures were conducted according to the manufacturer’s instructions. Microplate reader (Multiskan FC microplate photometer, Thermo Scientific, Waltham, MA, USA) was used to measure optical density at a wavelength of 450 nm. The SIRT1 level was calculated by using the standard curve. The sensitivity range of the standard curve was 0.63–40 ng/mL. The sensitivity range of the standard curve was 132 pg/mL.

### 2.4. Statistical Analysis

Statistical analysis was conducted with SPSS/W 20.0 software (Statistical Package for the Social Sciences for Windows, Inc., Chicago, IL, USA). Gender distribution was presented as absolute numbers with percentages and compared with the χ^2^ test. Continual data (age and serum SIRT1 level) were introduced as median with interquartile range (IQR). Data which not ordinally distributed between the two subjects’ groups or subgroups were measured with the Mann Whitney U test.

We conducted a Hardy-Weinberg analysis to analyze the observed and expected frequencies of *SIRT1* rs3818292, rs3758391, and rs7895833 with the χ^2^ test in the control group. The *SIRT1* rs3818292, rs3758391, and rs7895833 between the studied groups with early and exudative AMD and the controls were analyzed with the χ^2^ test. In addition, binary logistic regression analysis was performed to assess the influence of genotypes on the development of early and exudative AMD. Odds ratios (OR) and 95% confidence intervals (CI) are reported. The Akaike Information Criterion (AIC) selected the best genetic model. Statistically significant differences were considered when *p* < 0.05. We also presented an altered significance threshold for multiple comparisons alpha = 0.017 (0.05/3 since we assessed three SNPs in the *SIRT1* gene)

In addition, we performed haplotype association analysis in different subjects’ groups: the early AMD and control groups and exudative AMD and control groups separately. It was performed using online SNPStats website (https://www.snpstats.net/snpstats/) (accessed on 22 April 2022). Website was created in 2006, it is a property of the Catalan Institute of Oncology (Barcelona, Spain) [28] linkage disequilibrium (LD) was measured using D‘ and r^2^. The associations between the haplotypes and early or exudative AMD were calculated using logistic regression. The associations were introduced as ORs and 95% CI and measures adjusted by age in exudative AMD examination. All haplotypes with frequencies less than 1% were pooled into the same group and presented as rare haplotypes.

## 3. Results

We performed genotyping of three SNPs of the *SIRT1* gene (rs3818292, rs3758391, and rs7895833). This examination was conducted in three groups, including patients with early AMD (*n* = 308), patients with exudative AMD (*n* = 411), and the control group (*n* = 225).

No statistically significant deviation was found in the genotype and allele distribution of the tested SNPs from Hardy-Weinberg equilibrium (HWE) (*p* > 0.05). The study’s control group was formed according to the sex and age distribution in early AMD and sex in the exudative AMD group. Hardy-Weinberg equilibrium (HWE) analysis was performed to evaluate the distribution of genotypes in controls. The study showed that all three SNPs met the HWE criteria (*p* > 0.05).

Females accounted for 68.5% (*n* = 211) of the early AMD group, 64.7% (*n* = 266) of the exudative AMD, and 64.7% (*n* = 266) of the control group. The control group was younger than the patients with exudative AMD (*p* = 0.008) (Table 2), but patients were adjusted for age in further statistical analysis. The median ages of the patients with early AMD, exudative AMD, and control subjects were 74 years (IQR = 12), 77 years (IQR = 10), and 74 years (IQR = 7), respectively.

The genotype and allele distributions of *SIRT1* genetic variants rs3818292 and rs7895833 were significantly different between early AMD and the control group and between exudative AMD and the control group after strict Bonferroni correction. The rs3818292 G allele is more frequent in the exudative AMD group than in the control group (9.5% vs. 5.6%, *p* = 0.014, respectively).

The rs7895833 G allele is more common in early and exudative AMD than in the control group (13.1% vs. 12.9% and 18.5% vs. 12.9%, *p* = 0.007 and *p* = 0.010, respectively). After additionally conducted comparation between early and exudative AMD, we found that *SIRT1* rs7895833 AA, AG and GG genotype distributions are also statistically significantly different (75.0%, 23.7% and 1.3% vs. 66.9%, 29.2%, 3.9%, *p* = 0.019). The rs7895833 A allele is more common in early AMD than in exudative AMD (86.9% vs. 81.5%, *p* = 0.007) (Table 3).

In addition, we performed binary logistic regression analysis to evaluate the effects of these SNPs on early and exudative AMD. The analysis revealed that the rs3818292 AG genotype is associated with a 1.8-fold and 1.7-fold increased odds of developing exudative AMD under the codominant model (OR = 1.750; CI: 1.073–2.855; *p* = 0.025) and under the overdominant model (OR = 1.735; CI: 1.064–2.829; *p* = 0.027), respectively. Unfortunately, these results did not survive strict Bonferroni correction. In addition, the AG+GG genotypes are associated with 1.8-fold increased odds of developing exudative AMD according to the dominant model (OR = 1.823; CI: 1.120–2.966; *p* = 0.016). Each G allele increases the odds of developing exudative AMD by 1.9-fold under the additive model (OR = 1.845; CI: 1.148–2.966; *p* = 0.011).

According to the codominant model, the *SIRT1* rs3758391 TT genotype is associated with a 2.2-fold increase in the odds of developing exudative AMD (OR = 2.226; CI: 1.039–4.942; *p* = 0.040). According to the additive model, each T allele increased the odds ratio of developing exudative AMD by 1.4-fold according to the additive model (OR = 1.367; CI: 1.037–1.800; *p* = 0.026).

The *SIRT1* rs7895833 GG genotype is associated with 5.3-fold (OR = 5.246; CI: 1.187–23.196; *p* = 0.029), AG+GG genotypes with 1.5-fold (OR = 1.538; CI: 1.064–2.223; *p* = 0.022) and GG with a 4.8-fold (OR = 4.756; CI: 1.080–20.939; *p* = 0.039) increased odds of exudative AMD according to the codominant, dominant, and recessive models, respectively. When we applied Bonferroni corrected significance threshold, these results did not reach statistical significance. On the other hand, each G allele at rs7895833 increases the odds of developing exudative AMD by 1.6-fold under the additive model (OR = 1.577; CI: 1.133–2.196; *p* = 0.007) (Table 4). No significant associations were found in the early AMD group (data not shown).

The pathogenesis of AMD can be distinguished by sex [1]; based on these data, we performed SNP analysis in men and women separately.

The analysis showed that the rs7895833 G allele is more common in women with exudative AMD than in the control women (18.8% vs. 11.8%, *p* = 0.011). Comparison between women with early and exudative AMD showed that *SIRT1* rs7895833 AA, AG and GG genotype distributions are also statistically significantly different (76.8%, 22.3%, 0.9% vs. 66.5%, 29.3%, 4.1%, *p* = 0.015). Also, the rs7895833 A allele is more common in women with early AMD than with exudative AMD (87.9% vs. 81.2%, *p* = 0.005). Our study also revealed that the rs3758391 C allele is more common in early AMD women than in exudative AMD (74.4% vs. 69.7%, *p* < 0.001). The *SIRT1* rs3818292 G allele is more common in early AMD than in controls (10.8% vs. 4.5%, *p* = 0.023), but this result did not reach Bonferroni corrected significance level; also, the same allele was more common in men with exudative AMD than in the controls (12.2% vs. 4.5%, *p* = 0.006, respectively) (Table 5).

Binomial logistic regression analysis in women revealed that *SIRT1* rs7895833 AG+GG genotypes are associated with a 1.7-fold increase in the odds of exudative AMD under the dominant model (OR = 1.729; CI: 1.073–2.786; *p* = 0.024), but this results did not reach statistical significance after Bonferroni correction. On the other hand, it was shown that each G allele increases the odds ratio of developing exudative AMD by 1.8-fold under the additive model (OR = 1.753; CI: 1.140–2.697; *p* = 0.011) (Table 6). No statistically significant associations were found in women with early AMD (data not shown).

Binomial logistic regression analysis in men showed that the genotypes rs3818292 AG and AG+GG are associated with a 2.5-fold (OR = 2.498; CI: 1.033–6.0416; *p* = 0.042) and 2.6-fold (OR = 2.630; CI: 1.094–6.323; *p* = 0.031) increased odds of early AMD according to the codominant and dominant genetic models, respectively. The results also showed that the rs3818292 genotype AG is associated with a 2.7-fold increase in the odds of exudative AMD according to the codominant, dominant, and overdominant genetic models (OR = 2.667; CI: 1.159–6.142; *p* = 0.021), and each allele G at rs3818292 is associated with a 2.7-fold increase in the odds of exudative AMD under the additive genetic model (OR = 2.667; CI: 1.159–6.142; *p* = 0.021) (Table 7). None of these results survived Bonferroni correction.

### 3.1. SIRT1 Haplotype Analysis

Haplotype analysis was performed in separate groups of AMD.

Pairwise linkage disequilibrium (LD) between studied polymorphisms was observed (Table 7).

Haplotype analysis did not show any associations with early AMD (Table 8) but revealed that individuals carrying rs3818292, rs3758391 and rs7895833 haplotype G-T-G had increased odds of exudative AMD (OR  =  2.05, 95% CI: 1.23–3.43; *p* = 0.0062) (Table 9) but the overall rare haplotypes were associated with the decreased odds of exudative AMD (OR  =  0.23, 95% CI: 0.06–0.89; *p* = 0.033) (Table 10).

### 3.2. SIRT1 Serum Levels in Early and Exudative AMD and Controls

**Figure 1:**
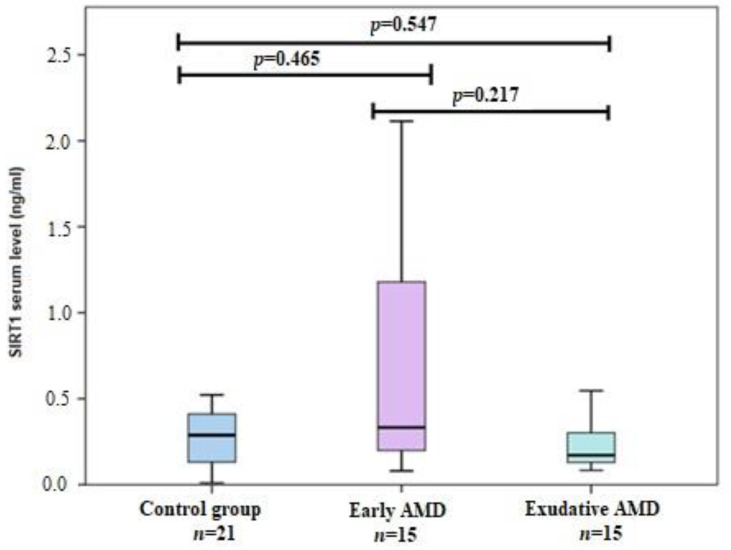
*SIRT1* serum levels between study groups.

We determined and compared *SIRT1* serum levels between early, exudative AMD and control groups but did not reveal significant differences between these groups (Figure 1).

*SIRT1* levels in early AMD (0.332 ng/mL (1.471)) vs. controls (0.288 ng/mL (0.343)), *p* = 0.465; *SIRT1* levels in exudative AMD (0.171 ng/mL (0.206)) vs. controls (0.288 ng/mL (0.343)), *p* = 0.547; *SIRT1* levels in early AMD (0.332 ng/mL (1.471)) vs. exudative AMD (0.171 ng/mL (0.206)), *p* = 0.217. Mann-Whitney U test was used to compare *SIRT1* levels between each two groups. The bars represent the median with interquartile range.

**Figure 2:**
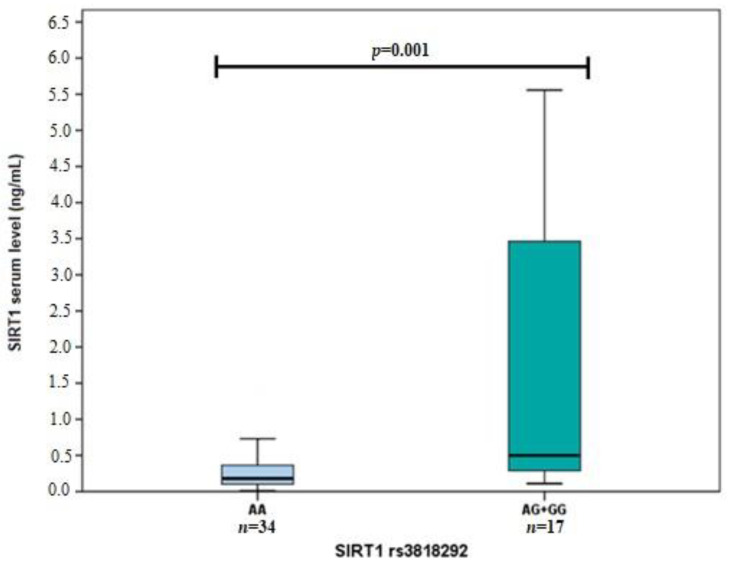
*SIRT1* serum level associations with *SIRT1* rs3818292.

**Figure 3:**
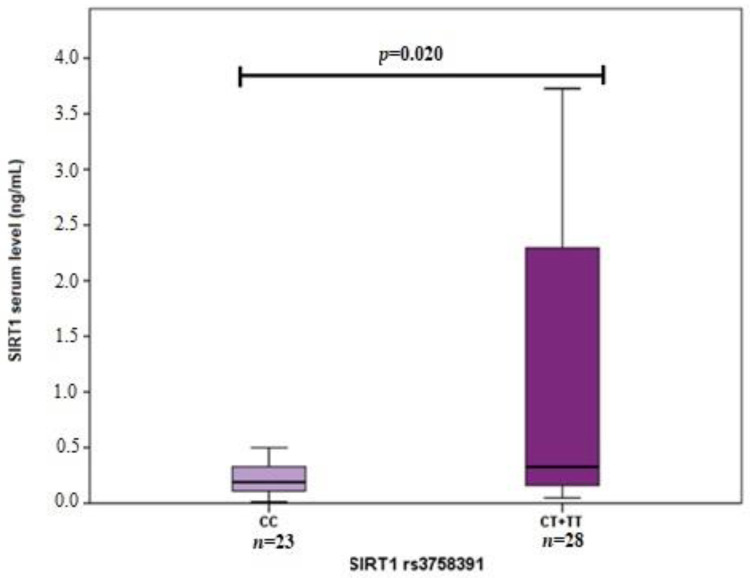
*SIRT1* serum level associations with *SIRT1* rs3758391.

**Figure 4:**
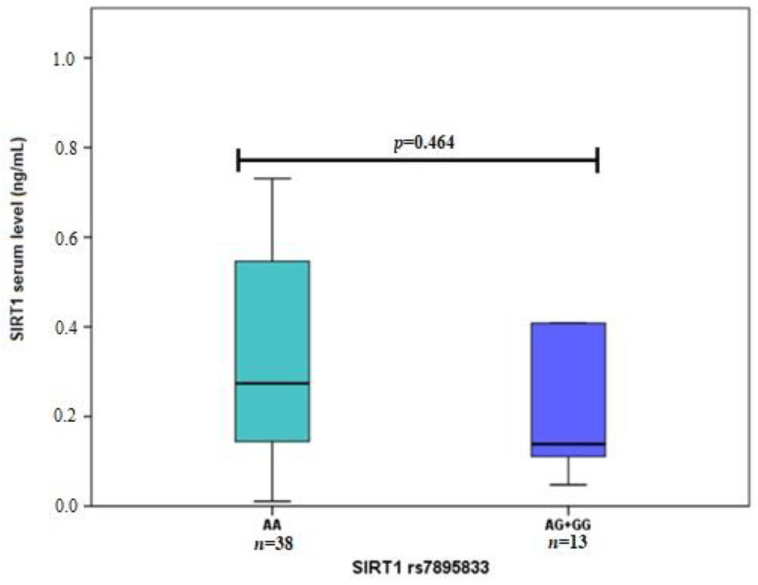
*SIRT1* serum level associations with *SIRT1* rs7895833.

Also, we performed *SIRT1* serum level and *SIRT1* genetic variant analysis. We found that *SIRT1* rs3818292 G allele carriers had higher *SIRT1* serum levels than the AA genotype (Figure 2). Moreover, higher *SIRT1* serum levels were determined for subjects carrying at least one *SIRT1* rs3758391 T allele than those with CC genotype (Figure 3). No associations were found between *SIRT1* rs7895833 and *SIRT1* levels (Figure 4).

*SIRT1* levels in subjects carrying at least one *SIRT1* rs3818292 G allele (0.499 ng/mL (3.310)) vs. subjects with AA genotype (0.180 ng/mL (0.267)), *p* = 0.001. Mann Whitney U test was used to compare *SIRT1* levels between two groups. The bars represent the median with an interquartile range.

*SIRT1* levels in subjects carrying at least one *SIRT1* rs3758391T allele (0.326 ng/mL (2.227)) vs. subjects with CC genotype (0.189 ng/mL (0.260)), *p* = 0.020. Mann Whitney U test was used to compare *SIRT1* levels between two groups. The bars represent the median with an interquartile range.

*SIRT1* levels in subjects carrying at least one *SIRT1* rs3758391 G allele (0.139 ng/mL (1.131)) vs. subjects with AA genotype (0.274 ng/mL (0.449)), *p* = 0.464. Mann Whitney U test was used to compare *SIRT1* levels between two groups. The bars represent the median with an interquartile range.

## 4. Discussion

Our study analyzed *SIRT1* gene polymorphisms rs3818292, rs3758391, and rs7895833 in AMD patients (*n* = 719) and healthy controls (*n* = 225). We found that rs3818292, rs3758391, and rs7895833 were associated with an increased risk of developing exudative AMD. However, we did not find significant results in the early AMD group. Additional sex-differentiated analysis revealed that *SIRT1* rs3818292 was associated with an increased risk of early and exudative AMD in men, and rs7895833 was associated with an increased risk of developing exudative AMD in women. However, no statistically significant associations were found in women with early AMD. Further analysis revealed that individuals carrying rs3818292, rs3758391 and rs7895833 haplotype G-T-G had increased odds of exudative AMD, but the rare haplotypes were associated with the decreased odds of exudative AMD.

After performing an analysis of serum *SIRT1* levels and *SIRT1* genetic variant, we found that carriers of the *SIRT1* rs3818292 minor allele G had higher serum *SIRT1* levels than the AA genotype. In addition, individuals carrying at least one *SIRT1* rs3758391 T allele also had elevated serum *SIRT1* levels compared with individuals with the wild-type CC genotype. While the intronic rs3818292 variant can affect the gene splicing processes and rs3758391 is a functional variant located in the promoter region, we believe that those variants led to the altered *SIRT1* protein expression, which was found comparing different genotype carriers and their serum levels.

As we mentioned earlier, AMD is characterized either by the presence of drusen (dry AMD) or by vascular epithelial growth factor (VEGF)-induced proliferation of choroidal endothelial cells with associated leakage (exudative AMD) [29]. In addition, two main pathological problems, oxidative stress and hypoxia, cause various pathological changes in the retina, including apoptotic cell death, retinal pigment epithelial cell dysfunction, lipofuscin accumulation, drusen formation, and Bruch’s membrane impairment [30,31].

*SIRT1* deacetylates and activates HIF-2α and regulates the vascular endothelial growth factor A (VEGF-A) promoter [32]. VEGF and fibroblast growth factor (FGF) promote angiogenesis [33]. At the same time, it is inhibited by pigment epithelium-derived factor (PEDF), angiostatin, endostatin, and others. Neovascularization is mainly associated with tissue ischemia, leading to increased secretion of VEGF and higher expression of VEGF-A2. VEGF causes vasodilation, leading to increased vascular permeability and protease activity. These changes allow the development and expansion of the vascular network in surrounding tissues and its remodeling [34]. The basilar membrane and intracellular connective tissue fragmentation are essential for forming new capillaries [20], and this mechanism was demonstrated with *SIRT1* in another experiment. The authors demonstrated that VEGF-A is regulated by *SIRT1* on hypoxic choroidal endothelial cells through the activation of HIF-2α [35].

Bhattacharya et al. [36] showed that P53 acetylation could increase Lys379 in primary human RPE by *SIRT1* inhibition. RPE cell apoptosis can be prevented by inhibiting p53 phosphorylation or acetylation. Another scientist group examined *SIRT1* expression in excised human choroidal neovascular membranes and donors’ eyes without AMD by immunohistochemistry and found increased *SIRT1* levels in human choroidal neovascular membranes compared with control eyes [37]. In an experimental animal model, *SIRT1* conditional knock out mice exhibit p53 hyperacetylation and decreased retinal neuronal cell numbers during development [38]. Jaliffa et al. showed that *SIRT1* is expressed in the mouse cornea, lens, iris, ciliary body, inner nuclear layer, outer nuclear layer, and retinal ganglion cell layer [39]. *SIRT1*-deficient mice are smaller than average at birth; they cannot open their eyes and usually die in the early postnatal period. [40]. Few studies have reported the significant role of *SIRT1* cataracts [41,42] and retinal degeneration development [43,44]. Moreover, it was reported that resveratrol could upregulate *SIRT1* in retinal cells and protect cells from apoptosis induced by an anti-retinal antibody [43].

In contrast, downregulation of *SIRT1* causes retinal damage by various mechanisms [45,46]. These results suggest that *SIRT1* protects the retina and optic nerve from degeneration [47].

Serum-induced *SIRT1* expression was associated with height, weight, % body fat and lean mass, albumin, and cholesterol but not with the disease. On the other hand, three *SIRT1* SNPs: rs2273773, rs3740051, and rs3758391, showed associations with weight, height, body fat, lean mass, and albumin levels. Only weak associations between *SIRT1* SNPs and arthritis, myocardial infarction, deafness, and cognitive impairment were determined. In conclusion, *SIRT1* SNPs and serum-induced *SIRT1* expression in older men may be more strongly related to diet and body composition than ageing and age-related diseases since scientists did not reveal associations between *SIRT1* SNPs serum-induced *SIRT1* assay (as well [48]).

Our previous study analyzed associations between *SIRT1* rs12778366 and AMD, but no statistically significant results were revealed [49]. On the other hand, controversial results were found in the Chinese Han population. Researchers revealed that homozygous carriers of the risk allele C of this SNP are associated with a higher risk of AMD development [47].

Conducted study shows a significant association between the *SIRT1* polymorphisms rs3818292 and rs7895833 and the development of exudative AMD with possible differences in females and males. Also, rs3818292, rs3758391, and rs7895833 haplotype G-T-G are associated with the exudative AMD development. Moreover, we revealed the associations between *SIRT1* promoter variants (rs3818292 and rs3758391) and elevated *SIRT1* serum levels. These results may be helpful for future studies to select prognostic factors for AMD or identify and develop new therapies to improve the daily life of AMD patients. However, further studies are needed to investigate the role of ageing and AMD development and the associations with *SIRT1*. It should be noted that our study has several limitations. First, our statistical analysis did not include other risk factors such as smoking, alcohol consumption, weight or diet preferences. Second, the response to the exudative AMD treatment was not included in the analysis. Finally, our study includes only the Lithuanian population, so it is limited with respect to the generalizability of its conclusions to other populations.

## 5. Conclusions

Our study showed that the *SIRT1* polymorphisms rs3818292, and rs7895833 and rs3818292-rs3758391-rs7895833 haplotype G-T-G could be associated with the development of exudative AMD and two SNPs (rs3818292 and rs3758391) are associated with elevated *SIRT1* levels.

## Figures and Tables

**Table 1:** Characteristics of polymorphisms.

Gene Polymorphisms	Position	Chromosome’s Location	Molecular Significance
*SIRT1* rs3818292	Chr10:67907144 [21]	Intragenic region [21]	Alteration of gene-splicing processes [22]
*SIRT1* rs3758391	Chr10:67883584 [25]	Promoter region [23]	Initiation of gene transcription [24]
*SIRT1* rs7895833	Chr10:67863299 [26]	Promoter region [23]	Initiation of a gene transcription [24]

**Table 2:** Demographic characteristics of the study groups.

Characteristic	Group			*p*-Value
	Early AMD *n* = 308 *n* (%)	Exudative AMD *n* = 411 *n* (%)	Control Group *n* = 225 *n* (%)	
Gender				
Males	97 (31.5)	145 (35.3)	89 (39.6)	0.054 *
Females	211 (68.5)	266 (64.7)	136 (60.4)	0.285 **
Age, (years), median (IQR)	74 (12)	77 (10)	74 (7)	0.052 * 0.008 **

* early AMD vs. control group. ** exudative AMD vs. control group.

**Table 3:** Distribution of genotypes and alleles of *SIRT1* rs3818292, rs3758391, and rs7895833 in early and exudative AMD and control groups.

Genotype/Allele	Early AMD *n* = 308 *n* (%)	Exudative AMD *n* = 411 *n* (%)	Control Group *n* = 225 *n* (%)	HWE *p*-Value	*p*-Value
***SIRT1* rs3818292**					
AA	263 (85.4)	336 (81.8)	200 (88.9)	0.378	0.291 *
AG	43 (14)	72 (17.5)	25 (11.1)		0.040 **
GG	2 (0.6)	3 (0.7)	0 (0)		0.430 ***
Total	308 (100)	411 (100)	225 (100)		0.183 *
A	569 (92.4)	744 (90.5)	425 (94.4)		0.014 **
G	47 (7.6)	78 (9.5)	25 (5.6)		0.216 ***
***SIRT1* rs3758391**					
CC	163 (52.9)	202 (49.1)	126 (56.0)	0.148	0.631 *
CT	128 (41.6)	178 (43.3)	90 (40.0)		0.100 **
CC	17 (5.5)	31 (7.5)	9 (4.0)		0.427 ***
Total	308 (100)	411 (100)	225 (100)		0.394 *
C	454 (73.7)	582 (70.8)	342 (76)		0.047 **
T	162 (26.3)	240 (29.2)	108 (24)		0.226 ***
***SIRT1* rs7895833**					
AA	231 (75.0)	275 (66.9)	169 (75.1)	0.302	0.905 *
AG	73 (23.7)	120 (29.2)	54 (24.0)		0.024 **
GG	4 (1.3)	16 (3.9)	2 (0.9)		0.019 ***
Total	308 (100)	411 (100)	225 (100)		0.007 *
A	535 (86.9)	670 (81.5)	392 (87.1)		0.010 **
G	81 (13.1)	152 (18.5)	58 (12.9)		0.007 ***

AMD-age-related macular degeneration; *p*-significance level and Bonferroni corrected significance level when *p* = 0.05/3. * Early AMD vs. Control group. ** Exudative AMD vs. Control group. *** Early AMD vs. Exudative AMD. The bold format points out the statistically significantly different results.

**Table 4:** Binomial logistic regression analysis of exudative AMD and control groups.

Model	Genotype/Allele	OR (95% CI) *	*p*-Value	AIC
Exudative AMD				
***SIRT1* rs3818292**				
Codominant	AG vs. AA GG vs. AA	1.750 (1.073–2.855) -	0.025 -	820.322
Dominant	AG+GG vs. AA	1.823 (1.120–2.966)	**0.016**	820.047
Recessive	GG vs. AG+AA	-	-	-
Overdominant	AG vs. AA+GG	1.735 (1.064–2.829)	0.027	821.113
Additive	G	1.845 (1.148–2.966)	**0.011**	819.337
***SIRT1* rs3758391**				
Co-dominant	CT vs. CC TT vs. CC	1.270 (0.904–1.783) 2.226 (1.039–4.942)	0.169 0.040	822.709
Dominant	CT+TT vs. CC	1.358 (0.977–1.888)	0.069	822.956
Recessive	TT vs. CT+CC	2.031 (0.946–4.361)	0.069	822.614
Overdominant	CT vs. CC+TT	1.170 (0.839–1.631)	0.355	825.424
Additive	T	1.367 (1.037–1.800)	0.026	821.238
***SIRT1* rs7895833**				
Co-dominant	AG vs. AA GG vs. AA	1.405 (0.964–2.047) 5.246 (1.187–23.196)	0.077 0.029	818.971
Dominant	AG+GG vs. AA	1.538 (1.064–2.223)	0.022	820.890
Recessive	GG vs. AG+AA	4.756 (1.080–20.939)	0.039	820.161
Overdominant	AG vs. AA+GG	1.336 (0.918–1.943)	0.130	823.946
Additive	G	1.577 (1.133–2.196)	**0.007**	818.614

AMD, age-related macular degeneration; OR, odds ratio; AIC, Akaike information criteria; CI, confidence interval; *p*, significance level. Bonferroni corrected significance level when *p* = 0.05/3; * Odds adjusted for age in exudative AMD analysis. The bold format points out the statistically significantly different results.

**Table 5:** Distribution of genotypes and alleles of *SIRT1* rs3818292, rs3758391, and rs7895833 in early and exudative AMD and control groups between different genders.

Genotype/Allele	Early AMD *n* = 308 *n* (%)	Exudative AMD *n* = 411 *n* (%)	Control Group *n* = 225 *n* (%)	*p*-Value
Females				
***SIRT1* rs3818292**				
AA	186 (88.2)	221 (83.1)	119 (87.5)	0.692 *
AG	24 (11.4)	42 (15.8)	17 (12.5)	0.300 **
GG	1 (0.5)	3 (1.1)	0 (0.0)	0.271 ***
Total	211 (100)	266 (100)	136 (100)	0.629 *
A	402 (93.9)	484 (91)	225 (93)	0.353 **
G	26 (6.1)	48 (9)	17 (7)	0.089 ***
***SIRT1* rs3758391**				
CC	112 (53.1)	128 (48.1)	75 (55.1)	0.677 *
CT	90 (42.7)	115 (43,2)	53 (39.0)	0.339 **
CC	9 (4.3)	23 (8,6)	8 (5.9)	0.139 ***
Total	211 (100)	411 (100)	136 (100)	0.947 *
	314 (74.4)	371 (69.7)	203 (74.6)	0.146 **
C	108 (25.6)	161 (30.3)	69 (25.4)	<0.001 ***
***SIRT1* rs7895833**				
AA	162 (76.8)	177 (66.5)	105 (77.2)	0.977 *
AG	47 (22.3)	78 (29.3)	30 (22.1)	0.977 **
GG	2 (0.9)	11 (4.1)	1 (0.7)	0.015 ***
Total	211 (100)	411 (100)	136 (100)	0.899 *
A	371 (87.9)	432 (81.2)	240 (88.2)	0.011 **
G	51 (12.1)	100 (18.8)	32 (11.8)	0.005 ***
**Males**				
***SIRT1* rs3818292**				
AA	77 (79.4)	115 (79.3)	81 (91.0)	0.072 *
AG	19 (19.6)	30 (20.7)	8 (9.0)	0.072 **
GG	1 (1.0)	1 (1.0)	0 (0.0)	0.465 ***
Total	97 (100)	146 (100)	89 (100)	0.023 *
A	173 (89.2)	230 (87.8)	170 (95.5)	0.006 **
G	21 (10.8)	32 (12.2)	8 (4.5)	0.647 ***
***SIRT1* rs3758391**				
CC	51 (57.3)	74 (51.0)	51 (57.3)	0.077 *
CT	37 (41.6)	63 (43.4)	37 (41.6)	0.077 **
CC	1 (1.1)	8 (5.5)	1 (1.1)	0.626 ***
Total	97 (100)	146 (100)	89 (100)	1.000 *
C	139 (78.1)	211 (72.8)	139 (78.1)	0.197 **
T	39 (21.9)	79 (27.2)	39 (21.9)	0.197 ***
***SIRT1* rs7895833**				
AA	64 (71.9)	98 (67.7)	64 (71.9)	0.879 *
AG	24 (27.0)	42 (29.0)	24 (27.0)	0.879 **
GG	1 (1.1)	5 (3.4)	1 (1.1)	0.745 ***
Total	97 (100)	146 (100)	89 (100)	1.000 *
A	152 (85.4)	238 (82.1)	152 (85.4)	0.349 **
G	26 (14.6)	52 (17.9)	26 (14.6)	0.349 ***

AMD, age-related macular degeneration; *p*, significance level. Bonferroni corrected significance level when *p* = 0.05/3. * Early AMD vs. Control group ** Exudative AMD vs. Control group *** Early AMD vs. Exudative AMD. The bold format points out the statistically significantly different results.

**Table 6:** Binomial logistic regression analysis of early AMD, exudative AMD, and control groups in females.

Model	Genotype/Allele	OR (95% CI) *	*p*-Value	AIC
Exudative AMD				
***SIRT1* rs3818292**				
Codominant	AG vs. AA GG vs. AA	1.349 (0.735–2.479) -	0.334 -	514.698
Dominant	AG+GG vs. AA	1.446 (0.791–2.642)	0.231	514.665
Recessive	GG vs. AG+AA	-	-	-
Overdominant	AG vs. AA+GG	1.331 (0.725–2.444)	0.356	515.282
Additive	G	1.502 (0.846–2.667)	0.165	514.121
***SIRT1* rs3758391**				
Co-dominant	CT vs. CC TT vs. CC	1.282 (0.831–1.977) 1.742 (0.738–4.114)	0.262 0.206	515.754
Dominant	CT+TT vs. CC	1.340 (0.884–2.033)	0.168	514.247
Recessive	TT vs. CT+CC	1.556 (0.674–3.595)	0.300	515.021
Overdominant	CT vs. CC+TT	1.196 (0.785–1.824)	0.405	515.460
Additive	T	1.301 (0.929–1.822)	0.126	513.766
***SIRT1* rs7895833**				
Co-dominant	AG vs. AA GG vs. AA	1.563 (0.964–2.047) 6.878 (0.872–547.279)	0.072 0.067	510.122
Dominant	AG+GG vs. AA	1.729 (1.073–2.786)	**0.024**	510.884
Recessive	GG vs. AG+AA	6.072 (0.773–47.709)	0.086	511.457
Overdominant	AG vs. AA+GG	1.478 (0.910–2.400)	0.114	513.589
Additive	G	1.753 (1.140–2.697)	**0.011**	509.123

AMD, age-related macular degeneration; OR, odds ratio; AIC, Akaike information criteria; CI, confidence interval; *p*, significance level. Bonferroni corrected significance level when *p* = 0.05/3; * Odds adjusted for genders in exudative AMD analysis. The bold format points out the statistically significantly different results.

**Table 7:** Binomial logistic regression analysis of early AMD, exudative AMD, and control groups in males.

Model	Genotype/Allele	OR (95% CI) *	*p*-Value	AIC
Early AMD				
***SIRT1* rs3818292**				
Codominant	AG vs. AA GG vs. AA	2.498 (1.033–6.041) -	**0.042** -	255.749
Dominant	AG+GG vs. AA	2.630 (1.094–6.323)	**0.031**	254.436
Recessive	GG vs. AG+AA	-	-	-
Overdominant	AG vs. AA+GG	1.298 (0.767–2.197)	0.331	255.180
Additive	G	1.411 (0.851–2.338)	0.182	254.046
***SIRT1* rs3758391**				
Co-dominant	CT vs. CC TT vs. CC	1.027 (0.566–1.864) 8.000 (0.965–66.302)	0.350 0.054	255.640
Dominant	CT+TT vs. CC	1.211 (0.678–2.160)	0.518	259.088
Recessive	TT vs. CT+CC	7.910 (0.969–64.572)	0.054	253.647
Overdominant	CT vs. CC+TT	0.905 (0.504–1.627)	0.739	259.396
Additive	T	1.413 (0.860–2.321)	0.172	257.615
***SIRT1* rs7895833**				
Co-dominant	AG vs. AA GG vs. AA	1.005 (0.524–1.926) 1.855 (0.164–20.955)	0.988 0.617	261.243
Dominant	AG+GG vs. AA	1.039 (0.549–1.965)	0.907	259.493
Recessive	GG vs. AG+AA	1.853 (0.165–20.791)	0.617	259.243
Overdominant	AG vs. AA+GG	0.992 (0.518–1.898)	0.980	259.506
Additive	G	1.073 (0.598–1.926)	0.813	259.450
**Exudative AMD**				
***SIRT1* rs3818292**				
Codominant	AG vs. AA GG vs. AA	2.667 (1.159–6.142) -	**0.021** -	306.335
Dominant	AG+GG vs. AA	2.667 (1.159–6.142)	**0.021**	304.335
Recessive	GG vs. AG+AA	-	-	-
Overdominant	AG vs. AA+GG	2.667 (1.159–6.142)	**0.021**	304.335
Additive	G	2.667 (1.159–6.142)	**0.021**	304.335
***SIRT1* rs3758391**				
Co-dominant	CT vs. CC TT vs. CC	1.283 (0.737–2.234) 5.771 (0.694–47.986)	0.378 0.105	308.174
Dominant	CT+TT vs. CC	1.407 (0.816–2.426)	0.220	308.841
Recessive	TT vs. CT+CC	5.158 (0.630–42.206)	0.126	306.956
Overdominant	CT vs. CC+TT	1.173 (0.679–2.028)	0.567	310.030
Additive	T	1.501 (0.923–2.441)	0.102	307.606
***SIRT1* rs7895833**				
Co-dominant	AG vs. AA GG vs. AA	1.210 (0.664–2.205) 3.382 (0.382–29.988)	0.533 0.274	310.600
Dominant	AG+GG vs. AA	1.298 (0.722–2.333)	0.383	309.590
Recessive	GG vs. AG+AA	3.197 (0.364–28.116)	0.295	308.991
Overdominant	AG vs. AA+GG	1.167 (0.642–2.121)	0.612	310.101
Additive	G	1.344 (0.794–2.275)	0.271	309.117

AMD, age-related macular degeneration; OR, odds ratio; AIC, Akaike information criteria; CI, confidence interval; *p*, significance level an; Bonferronirrected significance level when *p* = 0.05/3; * Odds adjusted for genders in exudative AMD analysis. The bold format points out the statistically significantly different results.

**Table 8:** Linkage disequilibrium between every two *SIRT1* SNPs.

SNPs	Early AMD vs. Controls			Exudative AMD vs. Controls		
	D’	r^2^	*p*-Value	D’	r^2^	*p*-Value
**rs3818292**–**rs3758391**	**0.9302**	**0.1848**	0.0	0.9321	0.2032	0.0
**rs3818292**–**rs7895833**	0.9142	0.4037	0.0	0.9235	0.3799	0.0
**rs3758391**–**rs7895833**	0.9329	0.3848	0.0	0.6978	0.4869	0.0

SNP, single nucleotide polymorphism; D’ is the deviation between the expected haplotype frequency and the observed frequency [D’ scale: 0.1] R^2^ is the squared correlation coefficient of the haplotype frequencies [r^2^ scale: 0.1] *p*—significance level when *p* = 0.05. The bold format points out the statistically significantly different results.

**Table 9:** Associations between *SIRT1* haplotypes and risk of early AMD.

Haplotype	rs3818292	rs3758391	rs7895833	Frequency (%)			OR (95% CI)	*p*-Value
				Early AMD	Controls	Total		
1	A	C	A	73.17	74.44	73.65	1.00	---
2	A	T	A	13.34	11.96	12.8	1.14 (0.77–1.69)	0.51
3	G	T	G	7.29	4.8	6.25	1.51 (0.78–1.50)	0.13
4	A	T	G	5.67	6.78	6.13	0.85 (0.51–1.42)	0.53
rare	*	*	*	NA	NA	1.15	0.28 (0.07–1.09)	0.066

OR, odds ratio; CI, confident interval; *p*, significance level when *p* = 0.05; AIC, Akaike information criteria; NA, not applicable, rare—pooled haplotypes with frequencies <1%; *–allele of rare haplotype. The bold format points out the statistically significantly different results.

**Table 10:** Associations between *SIRT1* haplotypes and risk of exudative AMD.

Haplotype	rs3818292	rs3758391	rs7895833	Frequency (%)			OR (95% CI)	*p*-Value
				Exudative AMD	Controls	Total		
1	A	C	A	70.4	74.44	71.79	1.00	---
2	A	T	A	10.71	11.96	11.19	0.98 (0.66–1.45)	0.92
3	A	T	G	9.41	16.78	8.49	1.45 (0.93–2.27)	0.1
4	G	T	G	9.08	4.84	7.58	2.05 (1.23–3.43)	**0.0062**
rare	*	*	*	NA	NA	0.96	0.23 (0.06–0.89)	**0.033**

OR, odds ratio; CI, confident interval; *p*, significance level when *p* = 0.05; AIC, Akaike information criteria; NA, not applicable; rare—haplotypes with frequencies <1%. Significant *p* < 0.05 in bold. *—allele of rare haplotype.

## Data Availability

All data relevant to the study are included in the article.
